# Supplementary material for: The clinical characteristics and SARS‐CoV‐2 infection in children of acute hepatitis with unknown aetiology: A meta‐analysis and systematic review
Source: PLoS One. 2024 Dec 5;19(12):e0311772. doi: 10.1371/journal.pone.0311772 (PMC11620374; doi:10.1371/journal.pone.0311772)
Supplement: S1 Fig — (DOCX) [file pone.0311772.s001.docx]

# Supporting Figures 4

**Figures of Egger test.**


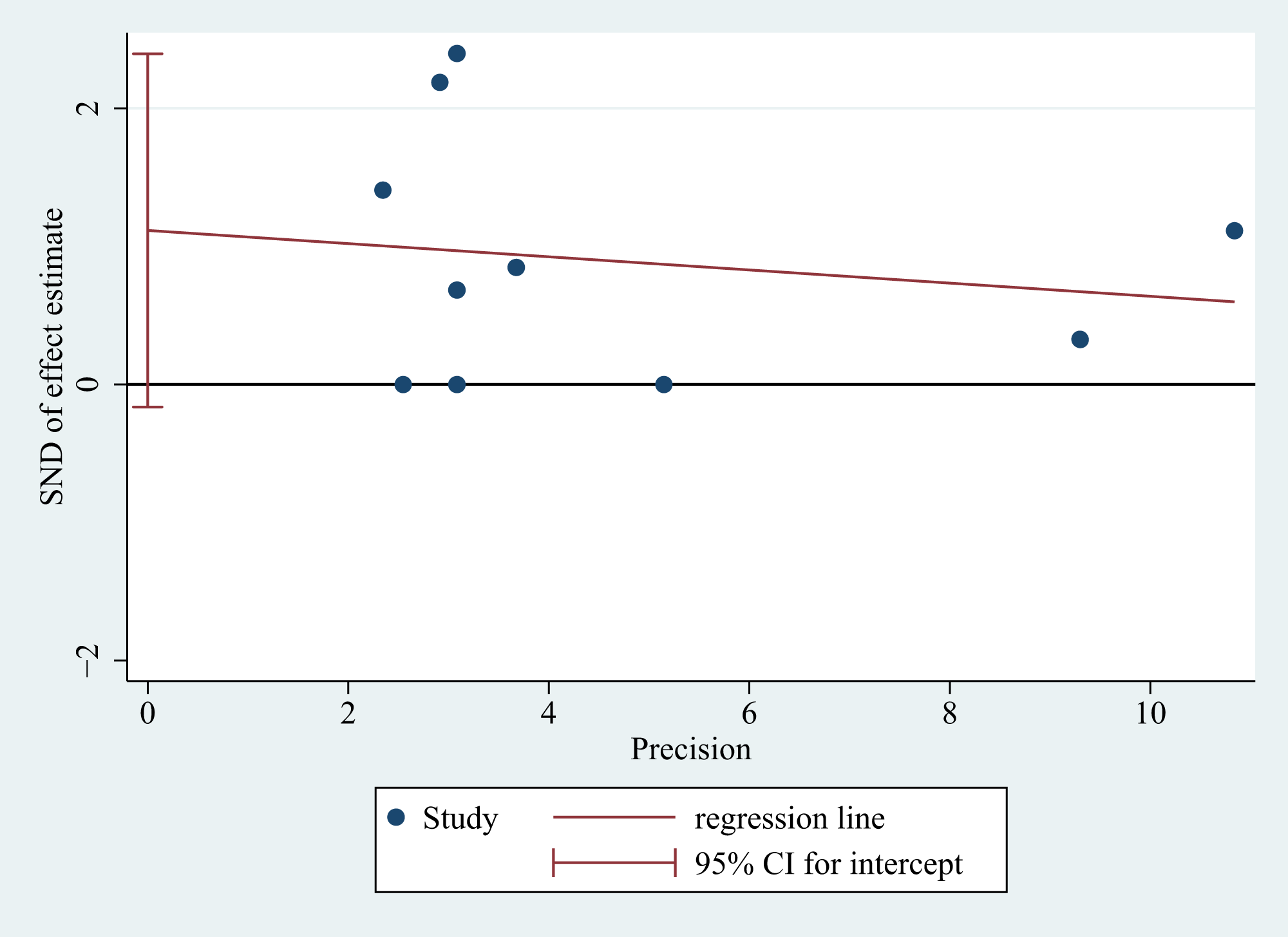


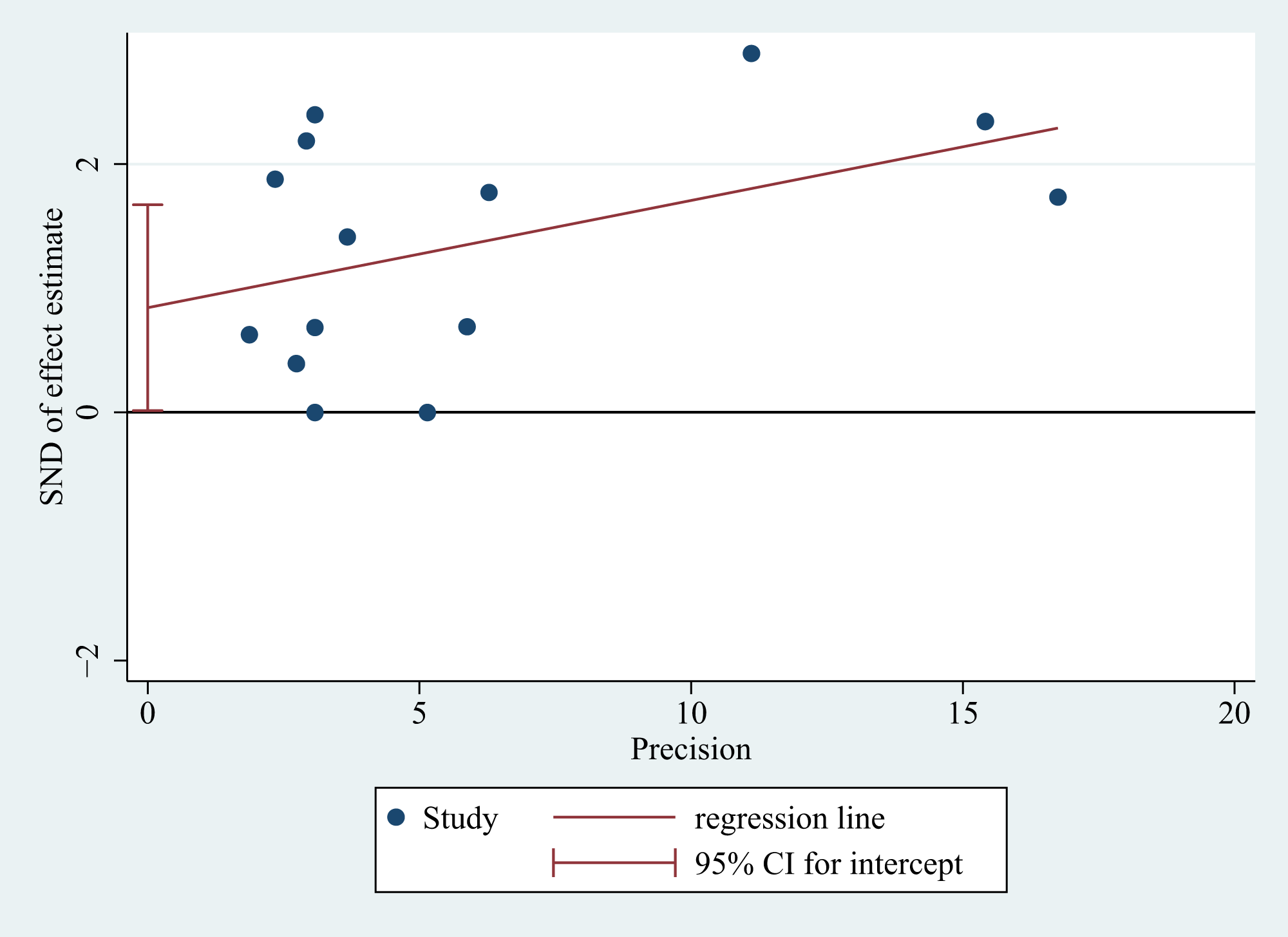


a.Egger test of SARS-CoV-2 infection. b. Egger test of previous infection of SARS-CoV-2.


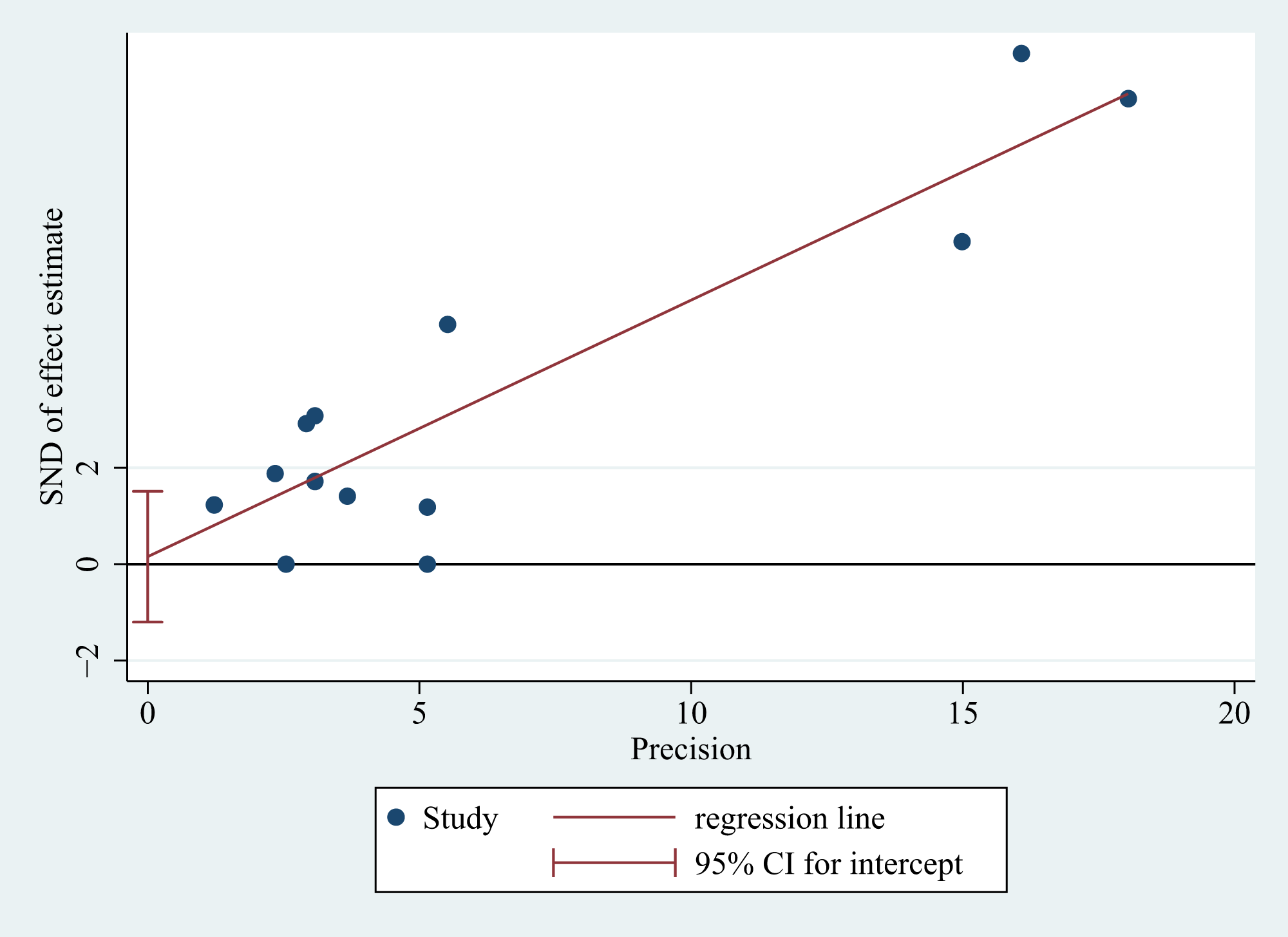

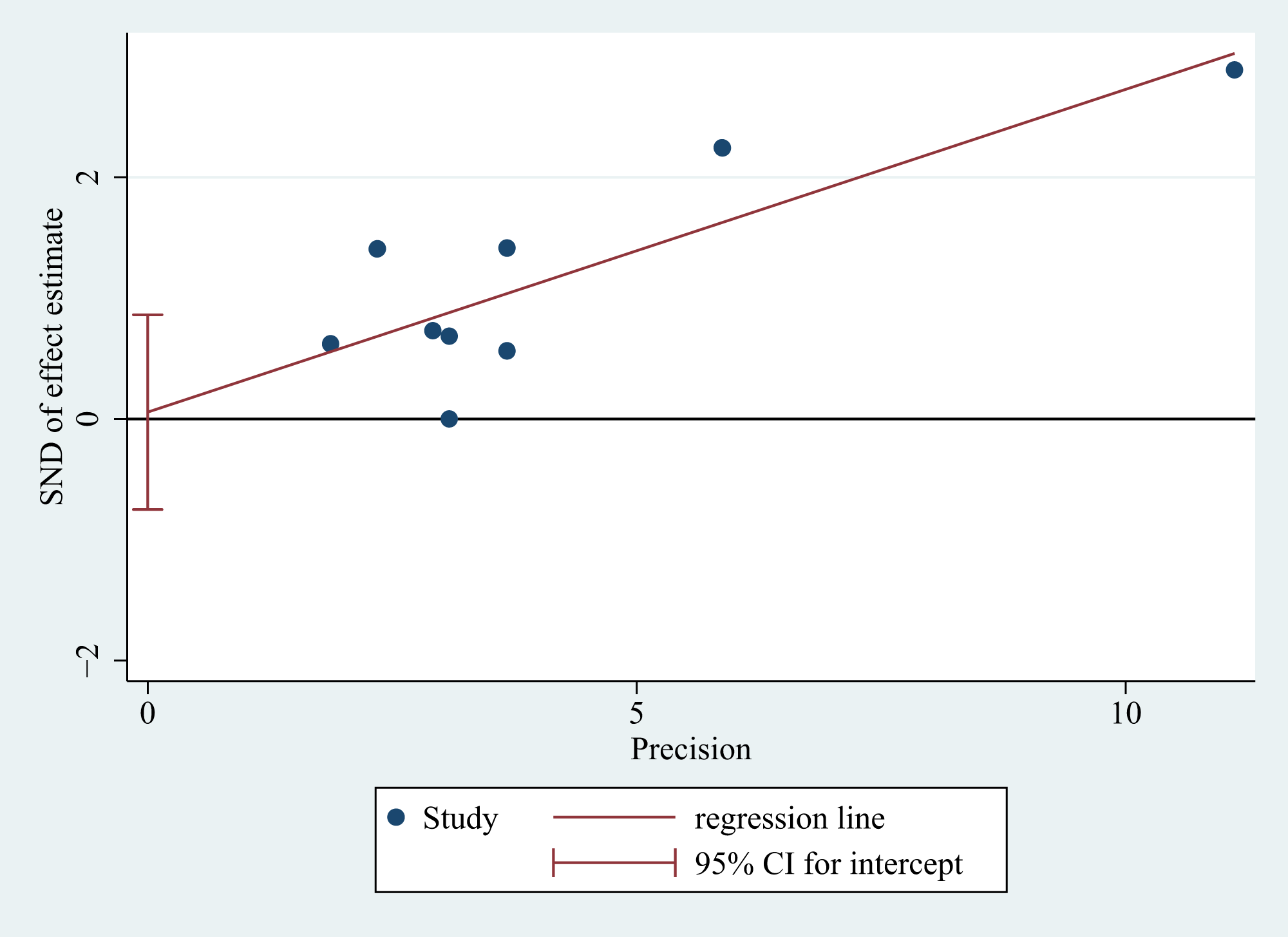


c. Egger test of SARS-CoV-2&adenovirus infection. d. Egger test of adenovirus infection.


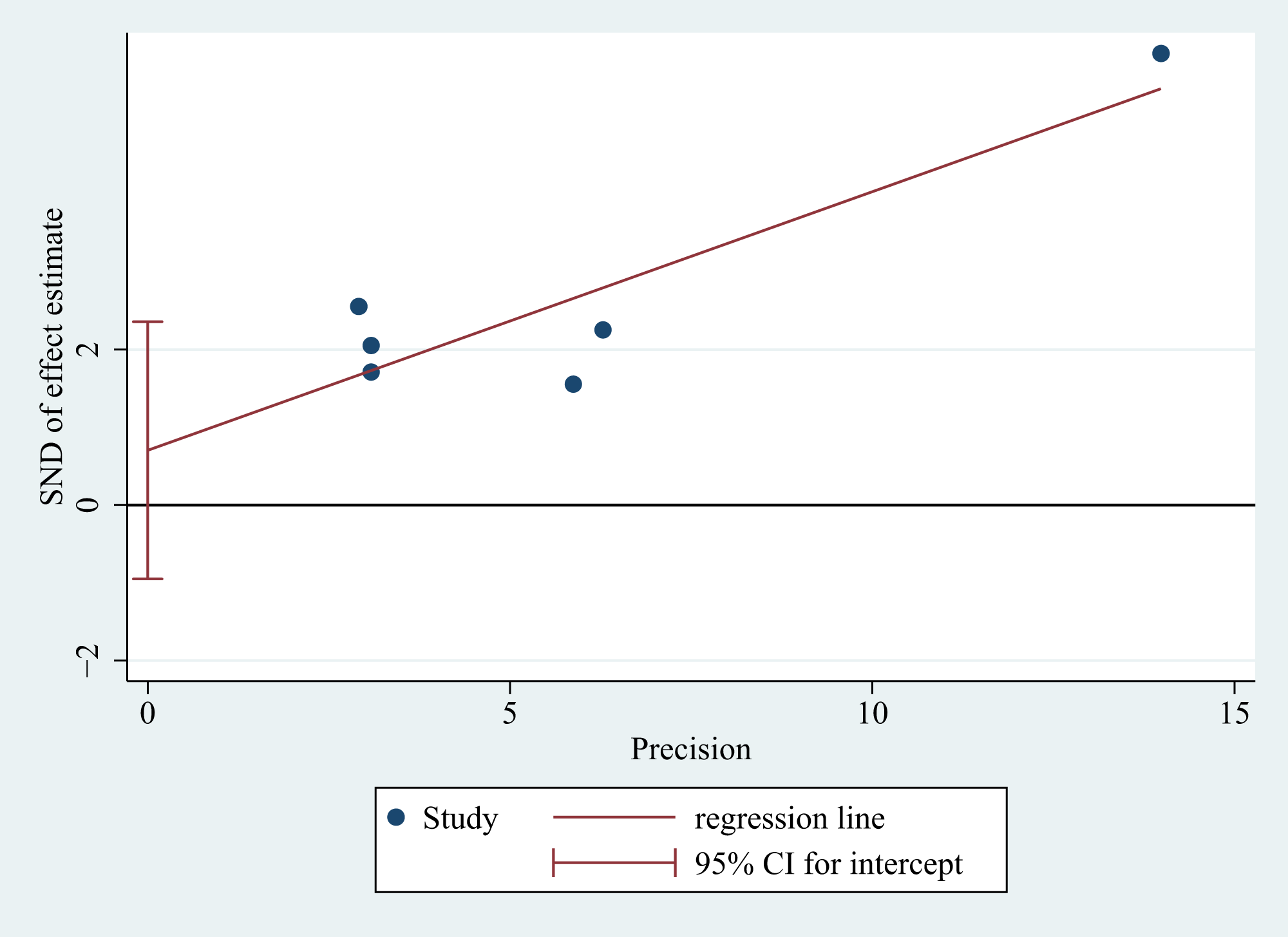

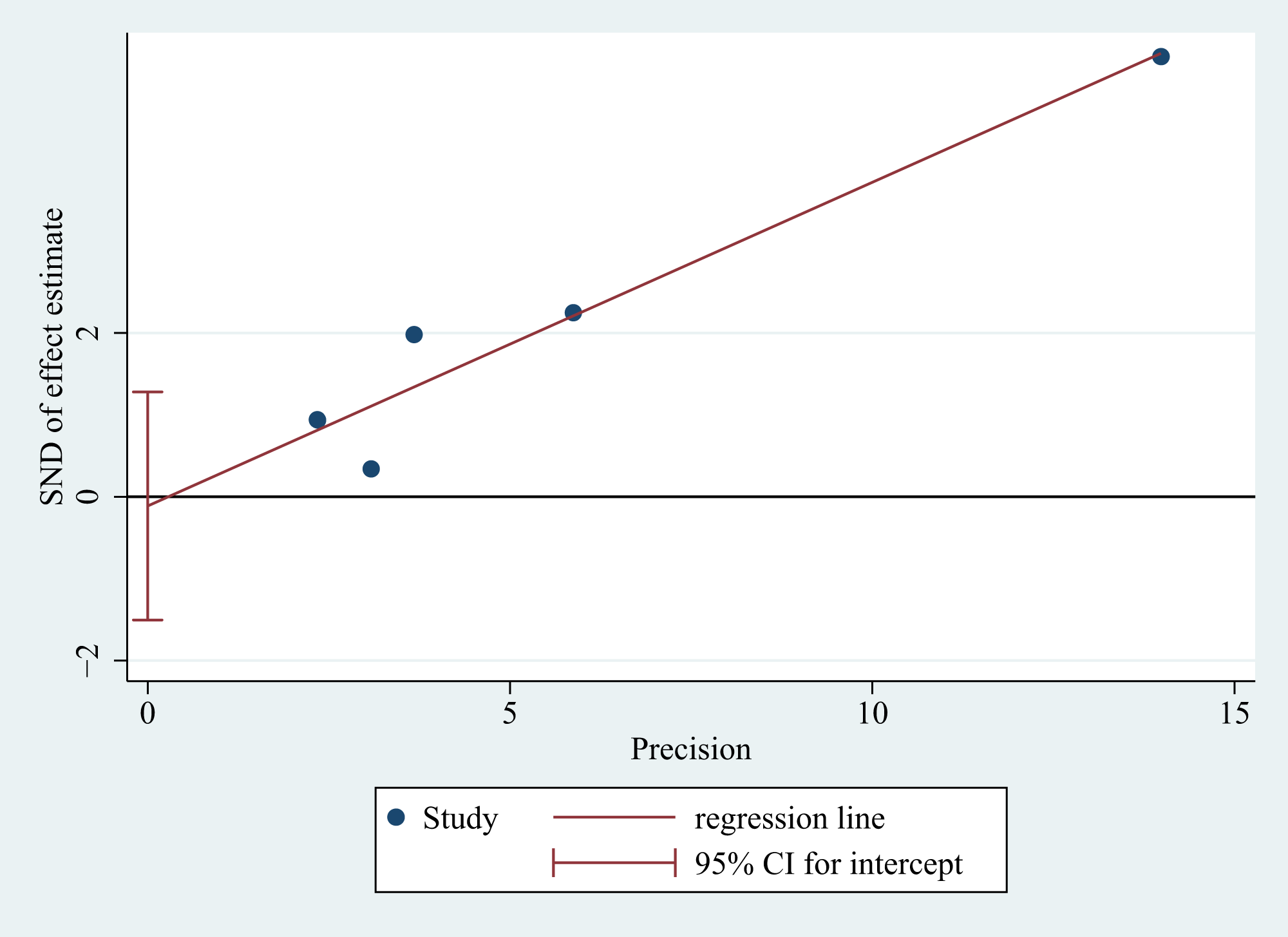


e. Egger test of abdominal pain. f. Egger test of diarrhea.


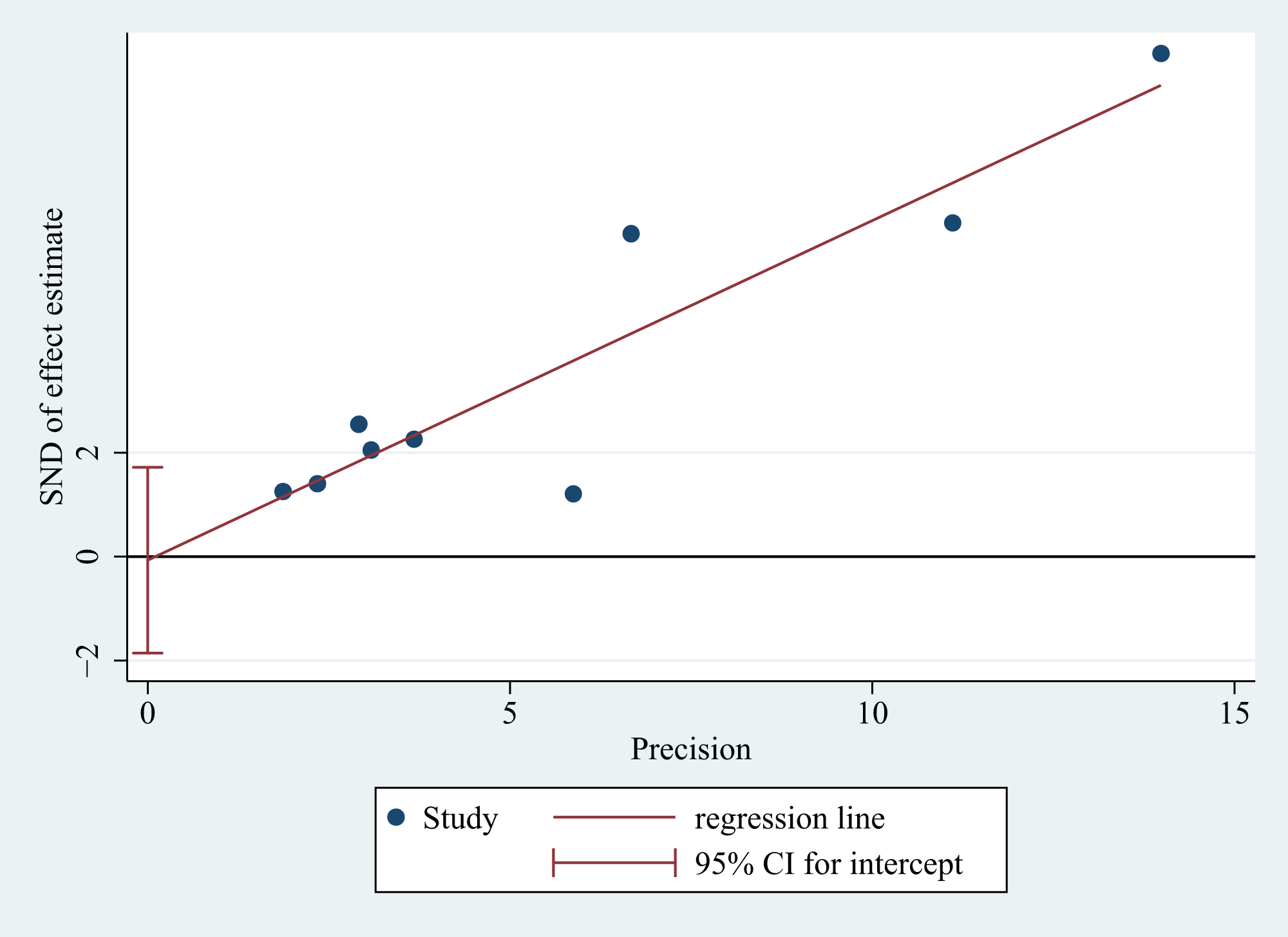

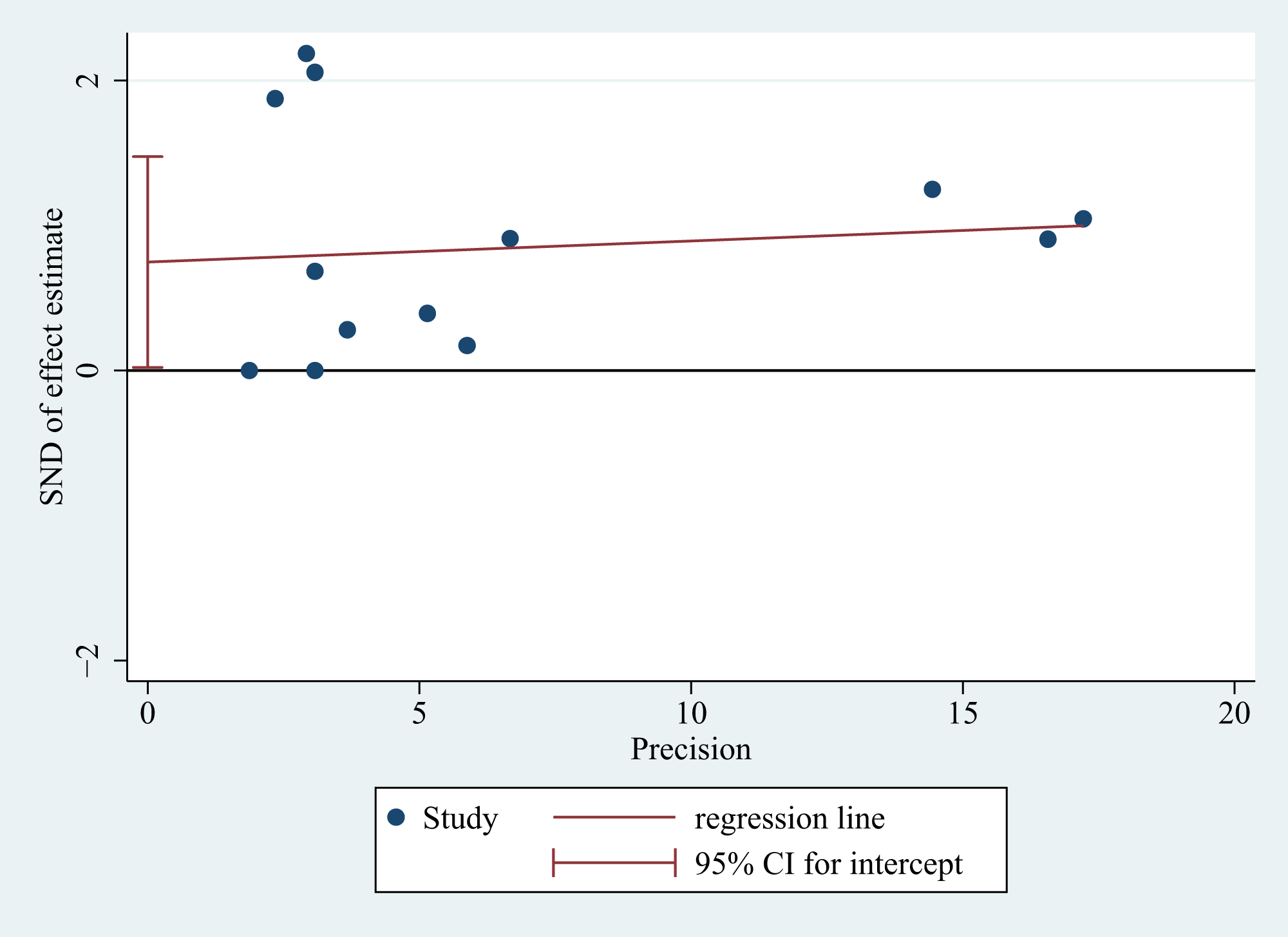


g. Egger test of liver transplantation. h.Egger test of jaundice.


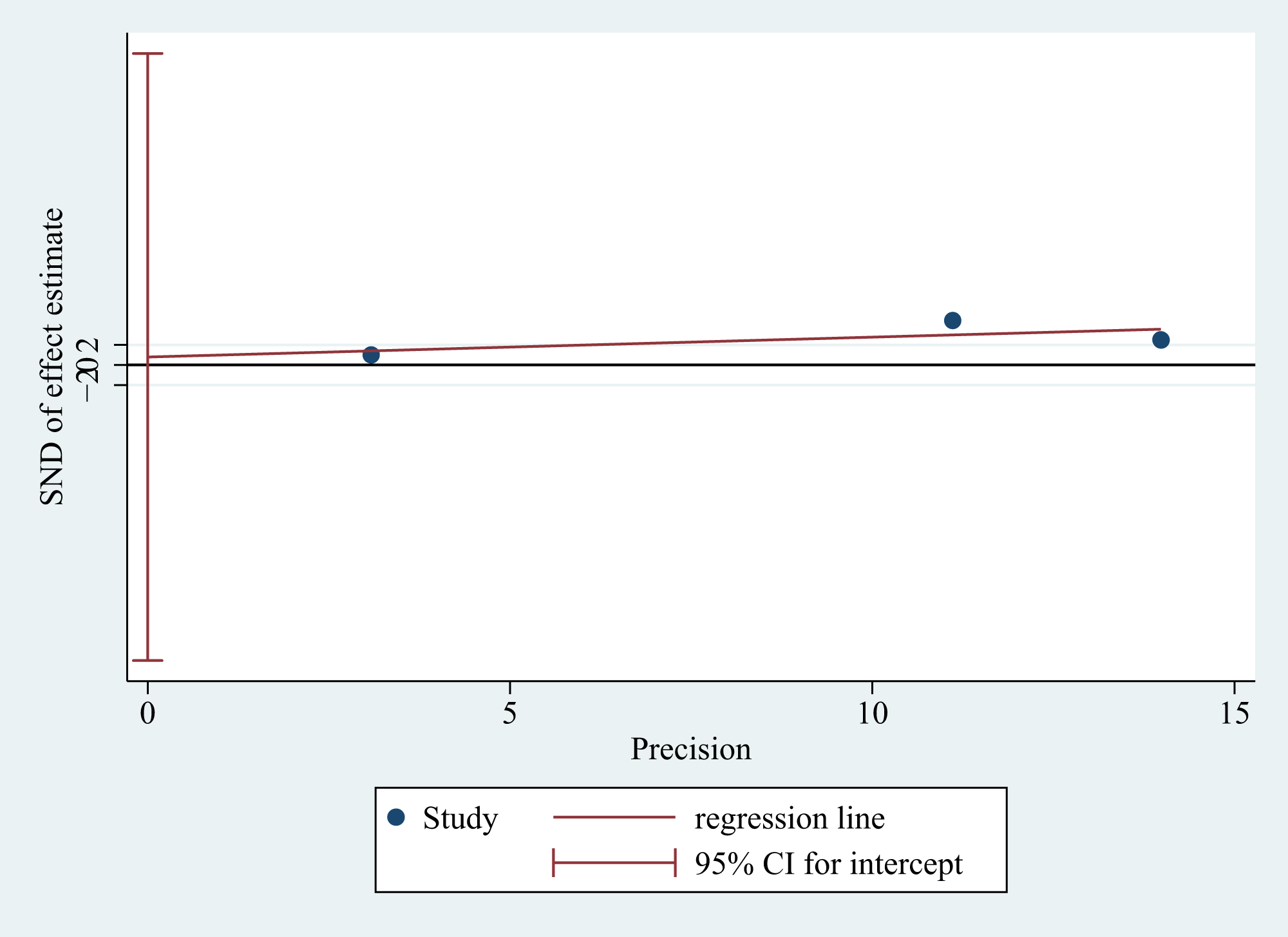

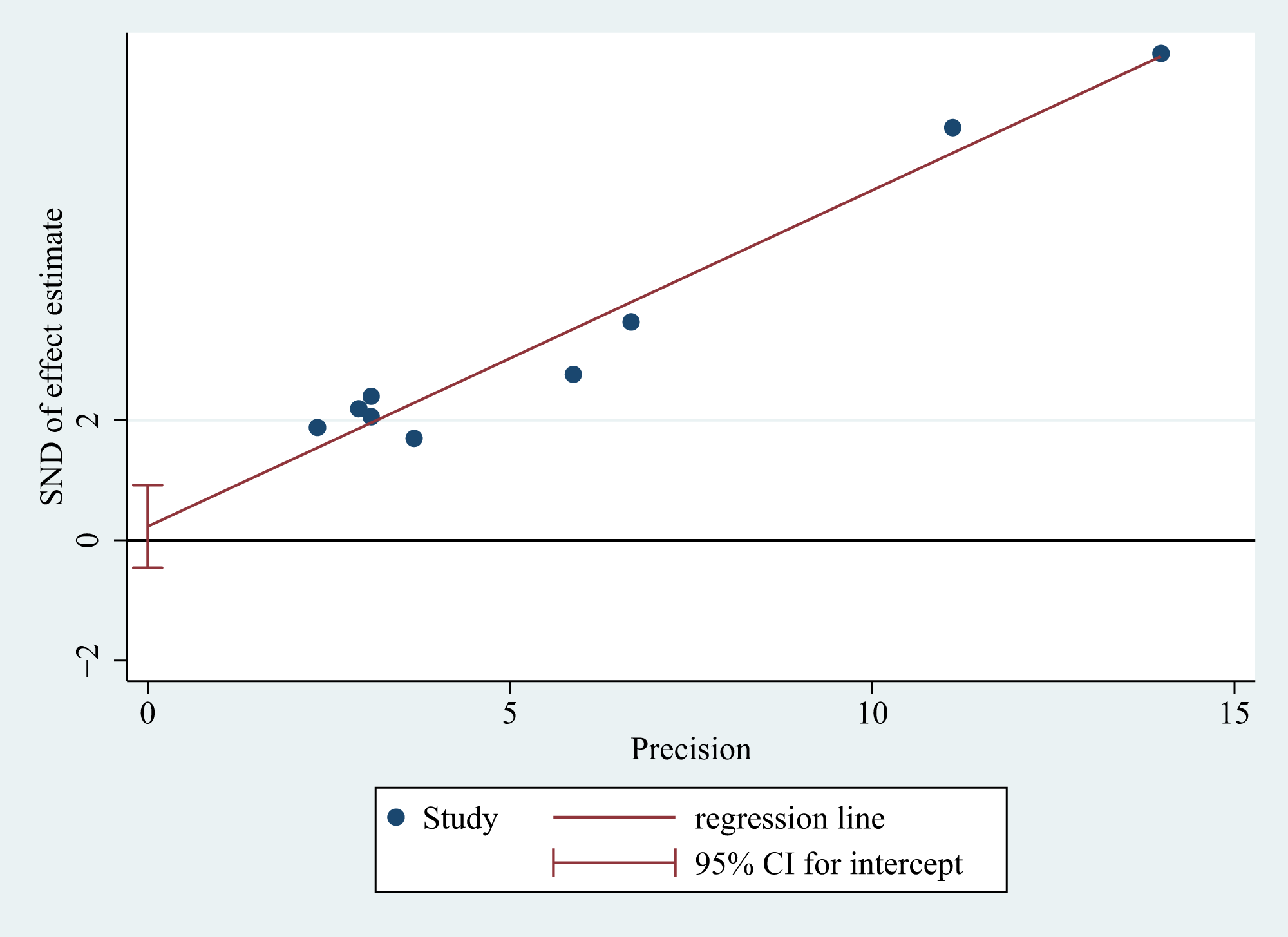


1. Egger test of vomiting. j. Egger test of upper respiratory symptoms.


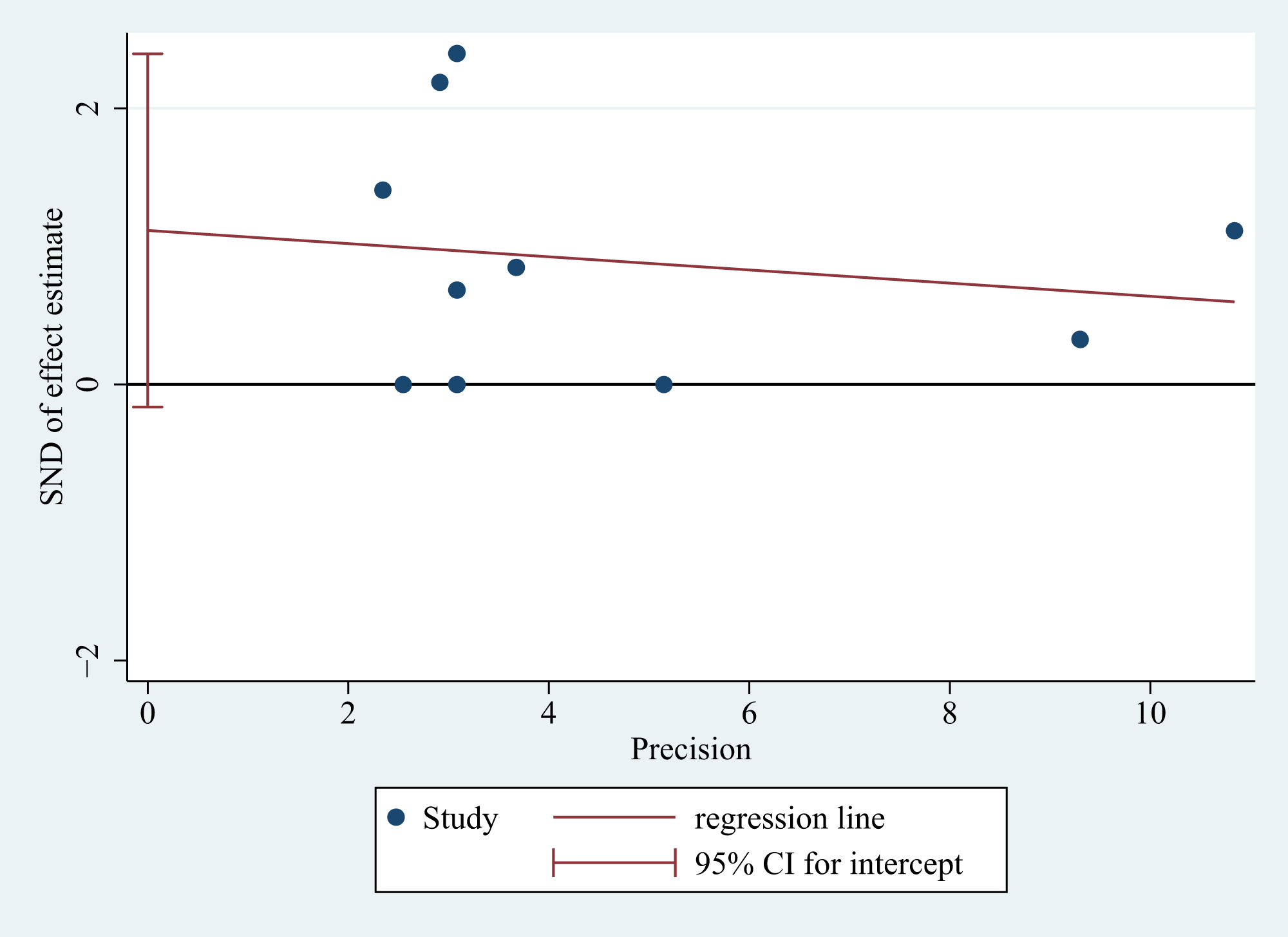


k.Egger test of fever.
