# Supplementary material for: The clinical characteristics and SARS‐CoV‐2 infection in children of acute hepatitis with unknown aetiology: A meta‐analysis and systematic review
Source: PLoS One. 2024 Dec 5;19(12):e0311772. doi: 10.1371/journal.pone.0311772 (PMC11620374; doi:10.1371/journal.pone.0311772)
Supplement: S1 Table — (DOCX) [file pone.0311772.s002.docx]

**Supporting 2 Table**

**Evaluation of articles by the Case Series Study Quality Assessment Tool.**

| Study | Q1 | Q2 | Q3 | Q4 | Q5 | Q6 | Q7 | Q8 | Total Score |
| --- | --- | --- | --- | --- | --- | --- | --- | --- | --- |
| Jordan C. | 0 | 1 | 1 | 1 | 0 | 1 | 1 | 1 | 6 |
| Willem S L. | 0 | 1 | 0 | 1 | 1 | 1 | 0 | 1 | 5 |
| Kimberly M. | 0 | 1 | 1 | 1 | 1 | 1 | 1 | 1 | 7 |
| Akash D. | 0 | 1 | 0 | 1 | 0 | 1 | 1 | 1 | 5 |
| Anita V. | 0 | 1 | 1 | 1 | 0 | 1 | 1 | 1 | 6 |
| Fabiola Di D. | 1 | 1 | 0 | 1 | 0 | 1 | 1 | 1 | 6 |
| Kelgeri C. | 0 | 1 | 1 | 1 | 1 | 1 | 1 | 1 | 7 |
| Adriana R V. | 1 | 1 | 0 | 1 | 0 | 0 | 1 | 1 | 5 |
| UKHSA | 1 | 1 | 0 | 1 | 0 | 0 | 1 | 1 | 5 |
| Julia M. B. | 0 | 1 | 1 | 1 | 1 | 1 | 1 | 1 | 7 |
| Pejman R. | 0 | 1 | 1 | 1 | 1 | 1 | 0 | 1 | 6 |
| Ruben H de K. | 1 | 1 | 0 | 0 | 0 | 0 | 1 | 1 | 4 |

Q1: Case series collected in more than one center? i.e. multi-center study.

Q2: Was the study question or objective clearly stated?

Q3: Was the study population clearly and fully described, including a case definition?

Q4: Were the cases consecutive?

Q5: Were the outcome measures clearly defined, valid, reliable and implemented consistently across all study participants?

Q6: Was the length of follow-up adequate?

Q7: Were the statistical methods well described?

Q8:Were the results well described?
