# Supplementary material for: The clinical characteristics and SARS‐CoV‐2 infection in children of acute hepatitis with unknown aetiology: A meta‐analysis and systematic review
Source: PLoS One. 2024 Dec 5;19(12):e0311772. doi: 10.1371/journal.pone.0311772 (PMC11620374; doi:10.1371/journal.pone.0311772)
Supplement: S2 Table — (DOCX) [file pone.0311772.s003.docx]

**Supporting 3 Table**

**Evaluation of articles by the Newcastle-Ottawa Quality Assessment Scale.**

| Study | Selection | Comparability | Exposure / Outcome | Total Score |
| --- | --- | --- | --- | --- |
| Elke W. | ★★★★ | ★★ | ★★ | 8 |
| Antonia H. | ★★★ | ★★ | ★★ | 7 |
