# Supplementary material for: The clinical characteristics and SARS‐CoV‐2 infection in children of acute hepatitis with unknown aetiology: A meta‐analysis and systematic review
Source: PLoS One. 2024 Dec 5;19(12):e0311772. doi: 10.1371/journal.pone.0311772 (PMC11620374; doi:10.1371/journal.pone.0311772)
Supplement: S1 File — (DOCX) [file pone.0311772.s004.docx]

# Supporting File 1

**Search strategy.**

Four databases including PubMed, Embase, Web of Science, and the Cochrane Library were systematically searched from 1 October 2021 to 8 December 2022.

**PubMed**

((((((((children) OR (pediatric)) OR (infant)) OR (baby)) OR (adolescent)) OR (teenager)) OR (juvenile)) OR (young people)) AND (((((((((((((pandemic) OR (Crisis)) OR (Epidemic)) OR (COVID-19)) OR (severe acute respiratory syndrome coronavirus 2)) OR (novel coronavirus)) OR (COVID-19)) OR (SARS-CoV-2)) OR (2019-nCoV)) OR (Covid)) OR (Omicron)) OR (SARS-CoV-2 variants)) AND (((((((((((((((((Acute hepatitis unknown origin) OR (Hepatitis Outbreak)) OR (Severe Acute Hepatitis Unknown Origin)) OR (unknown aetiology)) OR (acute liver failure)) OR (Acute hepatitis)) OR (novel pediatric hepatitis)) OR (novel hepatitis)) OR (inflammation liver)) OR (acute liver failure)) OR (liver transplants)) OR (Severe acute hepatitis)) OR (Transaminase)) OR (aspartate aminotransferase)) OR (AST)) OR (ALT)) OR (alanine aminotransferase)))

**Embase**

1# 'adolescent'/exp OR 'child'/exp OR 'Infant'/exp OR 'pediatric'/exp

2# 'Pandemics'/exp OR 'coronavirus disease 2019'/exp OR 'Severe acute respiratory syndrome coronavirus 2'/exp

3# 'Pandemics'/exp OR 'coronavirus disease 2019'/exp OR 'Severe acute respiratory syndrome coronavirus 2'/exp

4# 1# AND 2# AND 3#

**Web of Science**

1# TS=(adolescent OR Adolescents OR Adolescence OR Teens OR Teen OR Teenagers OR Teenager OR Youth OR Youths OR Adolescents, Female OR Adolescent, Female OR Female Adolescent OR Female Adolescents OR Adolescents, Male OR Adolescent, Male OR Male Adolescent OR Male Adolescents OR child OR children OR Infant* OR young children OR pediatric OR Bab*)

2# TS=(Pandemics OR Pandemic OR COVID* OR 2019-nCoV Infection OR 2019 nCoV Infection OR 2019-nCoV Infections OR Infection, 2019-nCoV OR SARS-CoV-2 Infection OR Infection, SARS-CoV-2 OR SARS CoV 2 Infection OR SARS-CoV-2 Infections OR 2019 Novel Coronavirus Disease OR 2019 Novel Coronavirus Infection OR COVID-19 Virus Infection OR COVID 19 Virus Infection OR COVID-19 Virus Infections OR Infection, COVID-19 Virus OR Virus Infection, COVID-19 OR COVID19 OR Coronavirus Disease 2019 OR Disease 2019, Coronavirus OR Coronavirus Disease-19 OR Coronavirus Disease 19 OR Severe Acute Respiratory Syndrome Coronavirus 2 Infection OR COVID-19 Virus Disease OR COVID 19 Virus Disease OR)

3# TS=(Hepatitis OR Hepatitides OR Liver Transplantation OR Grafting, Liver OR Liver Grafting OR Transplantation, Liver OR Liver Transplantations OR Liver Transplant OR Liver Transplants OR Transplant, Liver OR Hepatic Transplantation OR Hepatic Transplantations OR Transplantation, Hepatic OR Liver Failure OR Hepatic Failure OR Liver Failure, Acute OR Failure, Acute Liver OR Fulminant Hepatic Failure OR Fulminant Hepatic Failures OR Fulminating Hepatic Failure OR Fulminating Hepatic Failures OR Hepatic Failure, Fulminating OR Fulminating Liver Failure OR Fulminating Liver Failures OR Liver Failure, Fulminating OR Acute Liver Failure OR Liver Failure, Fulminant OR Fulminant Liver Failure OR Fulminant Liver Failures OR Hepatic Failure, Acute OR Acute Hepatic Failure OR Failure, Acute Hepatic OR Hepatic Failure, Fulminant OR etiology OR causality OR causes OR pathogenesis OR Hepatitis Outbreak OR unknown etiology OR acute liver failure OR Acute hepatitis OR novel hepatitis OR Liver inflammation OR Severe acute hepatitis)

4# 1# AND 2# AND 3#

**Cochrane Library**

1# adolescent OR Adolescents OR Adolescence OR Teens OR Teen OR Teenagers OR Teenager OR Youth OR Youths OR Adolescents, Female OR Adolescent, Female OR Female Adolescent OR Female Adolescents OR Adolescents, Male OR Adolescent, Male OR Male Adolescent OR Male Adolescents OR child OR children OR Infant* OR young children OR pediatric OR Bab*

2# Pandemics OR Pandemic OR COVID* OR 2019-nCoV Infection* OR 2019 nCoV Infection OR SARS-CoV-2 Infection* OR 2019 Novel Coronavirus Disease OR 2019 Novel Coronavirus Infection OR COVID-19 Virus Infection OR COVID 19 Virus Infection OR COVID-19 Virus Infections OR COVID19 OR Coronavirus Disease 2019 OR Coronavirus Disease-19 OR Coronavirus Disease 19 OR Severe Acute Respiratory Syndrome Coronavirus 2 Infection OR COVID-19 Virus Disease OR COVID 19 Virus Disease OR COVID-19 Virus Diseases OR SARS Coronavirus 2 Infection OR 2019-nCoV Disease OR 2019 nCoV Disease OR 2019-nCoV Diseases OR COVID-19 Pandemic OR COVID 19 Pandemic OR COVID-19 Pandemics OR SARS-CoV-2 OR SARS-CoV-2 Virus OR SARS CoV 2 Virus OR SARS-CoV-2 Viruses OR 2019 Novel Coronavirus OR 2019 Novel Coronaviruses OR COVID-19 Virus OR COVID 19 Virus OR COVID-19 Viruses OR Wuhan Coronavirus OR COVID19 Virus OR COVID19 Viruses OR Coronavirus Disease 2019 Virus OR Severe Acute Respiratory Syndrome Coronavirus 2 OR SARS Coronavirus 2 OR 2019-nCoV OR Wuhan Seafood Market Pneumonia Virus OR Crisis OR Epidemic OR Omicron OR severe acute respiratory syndrome coronavirus 2

3# Hepatit* OR Liver Transplantations OR Liver Transplant OR Liver Transplants OR Transplant, Liver OR Hepatic Transplantation OR Hepatic Transplantations OR Transplantation, Hepatic OR Liver Failure OR Hepatic Failure OR Liver Failure, Acute OR Failure, Acute Liver OR Fulminant Hepatic Failure OR Fulminant Hepatic Failures OR Fulminating Hepatic Failure OR Fulminating Hepatic Failures OR Hepatic Failure, Fulminating OR Fulminating Liver Failure OR Fulminating Liver Failures OR Liver Failure, Fulminating OR Acute Liver Failure OR Liver Failure, Fulminant OR Fulminant Liver Failure OR Fulminant Liver Failures OR Hepatic Failure, Acute OR Acute Hepatic Failure OR Failure, Acute Hepatic OR Hepatic Failure, Fulminant OR etiology OR causality OR causes OR pathogenesis OR Hepatitis Outbreak OR unknown etiology OR acute liver failure OR Acute hepatitis OR novel hepatitis OR Liver inflammation OR Severe acute hepatitis

4# 1# AND 2# AND 3#
