# Supplementary material for: The clinical characteristics and SARS‐CoV‐2 infection in children of acute hepatitis with unknown aetiology: A meta‐analysis and systematic review
Source: PLoS One. 2024 Dec 5;19(12):e0311772. doi: 10.1371/journal.pone.0311772 (PMC11620374; doi:10.1371/journal.pone.0311772)
Supplement: Attachment — Submitted filename: Rebuttal Letter.docx [file pone.0311772.s007.docx]

**October 8, 2023**

**Jiayi Shan**

**PLOS ONE**

**PONE-D-23-29634**

Dear Editors,

Thank you for your giving us an opportunity to revise our manuscript! Now we are submitting the revised manuscript entitled “The clinical characteristics and SARS‐CoV‐2 infection in children of acute hepatitis with unknown aetiology : A meta‐analysis and systematic review” for consideration for publication in ***PLOS ONE .***This manuscript is a resubmission of the manuscript PGPH-D-23-01412, which was previously considered by the journal PLOS Global Health Public.The Comments and responses are as follows:

（1）Reviewer #1:

**Comment 1:**I have attached my recommendation and I believe the manuscript is in good shape except some issues. The authors need to work on the method sections. In addition, the paper lacks key citations related meta analysis and scoping review.

**Response 1:**We have made all the necessary additions and modifications to the method sections, as shown in the red text.

（2）Reviewer #2:

**Comment 1:**This is a very relevant study for the scientific community, as there are still many doubts about the association between the COVID-19 pandemic and acute hepatitis with no defined etiology. ①However, the review needs to present the characteristics of the patients that were included in the meta-analysis and present a table with the quality of the studies included in the review. So that we can assess the relevance of the data presented in the review. ②I also missed that the authors further explored the pathogenesis between SARS-CoV-2 and adenoviruses for acute hepatitis. Since adenoviruses are ubiquitous viruses in nature, could the association with hepatitis not be a spurious association? I missed arguments that would support the association between adenovirus infection and acute hepatitis. Authors also need to review English. Some sentences are confused. Review the acronyms used and the formatting of the text.

**Response 1:①**We have completed the quality assessment of the studies in Supplemental material S2.②We have not yet found a definitive answer as to whether adenoviruses are involved in acute hepatitis, but the studies we have found suggest that the persistent immunosensitization of SARS-CoV-2 to the stinger protein superantigen in intestines, together with adenovirus intestinal infection, may lead to IFNγ release as well as IFNγ-mediated apoptosis of hepatocytes（seen in discussion section）.③Some grammatical corrections have been made throughout the text.

（3）Reviewer #3: The manuscript entitled The clinical characteristics and SARS‐CoV‐2 infection in children of acute hepatitis with unknown etiology : A meta‐analysis and systematic review is interesting. However, there are some important concerns that need to be addressed
**Comment 1:**It is better to submit the search strategy used in these four databases as a supplementary file.

**Response 1:**We have updated the search strategy as requested in Supplemental material S1.

**Comment 2:**The authors have used a specific checklist of case report or case series studies to evaluate the quality of all studies, while the type of studies in this article is not only case report and case series. Has the validity and reliability of this tool been proven for other types of studies in previous studies?

**Response 2:**For case control studies, we used the Newcastle-Ottawa Quality Assessment Scale for additional clarification, as detailed in Supplemental material S2.

**Comment 3:**Researchers have only reported the overall score obtained from the quality assessment tool, it is better to include a table including the item-by-item evaluation of the articles by this tool either in the article itself or in the supplementary file.

**Response 3:**We would like to apologise for not uploading the specific scores for the quality assessment tool in time, which have been added to Supplemental material S2.

**Comment 4:**It is better to specify the primary and secondary outcome in the method section.

**Response 4:**We have made additions to the primary and secondary outcomes, please see 2.4 in the revised manuscript for details, marked in red.

**Comment 5:**The quality of the figures, especially the forest plots, is very low, and the resolution of the images should be increased.

**Response 5:**We've resized all the images and all been re-uploaded.

**Comment 6:**The researchers did not report the heterogeneity in the obtained results, especially when the heterogeneity was high, it would have been better to consider subgroup analyses.

**Response 6:**We have previously identified high heterogeneity in SARS-CoV-2 infections and performed area subgroup analyses for this, but no cause for the heterogeneity was uncovered.Nevertheless, we added subgroup analyses by study types, and similarly no causes of heterogeneity were identified. However, all of our forest plots used random effects.（Figure 5）

**Comment 7:**In order to evaluate the publication bias, the results of Egger and Begg tests should be reported separately for each outcome.

**Response 7:** We conducted Egger tests on all the outcomes, and the results are shown in the Supplemental material S3 and Table 4.

**Comment 8:**The manuscript needs to be revised in terms of grammar.

**Response 8:**Some grammatical corrections have been made throughout the text.

（4）Reviewer #4:

**Comment 1:**The authors talk about meta-analysis and systematic. However, I want more examples and a paragraph about why they use this method and its benefits/limitations.

**Response 1:**Meta-analysis, which has come to be regarded as the highest level of evidence in evidence-based medicine. The results of multiple small-sample studies on the same topic can be synthesised to improve the statistical efficacy of the original results. Given that there are fewer randomised controlled trials and more cross-sectional studies on the association between acute hepatitis and SARS-CoV-2/adenoviruses, Meta-analysis can be performed first to point the way for further research.

**Comment 2:**Why does the author only use four databases? Why did they not expand their searches? I would like to see at least some justification for using only four databases.

**Response 2:**These four databases are the major databases that include acute hepatitis and provide a more comprehensive collection of studies used as Meta-analyses.

**Comment 3:** I would also recommend adding a figure or chart showing steps and narrowing down the search results. It can place either in the method or literature inclusion and characteristics section. See the Figure from Ahasan et al. (2022) study as a possible visualization example.

**Response 3:** We have added a flowchart to simply represent the entire search process and results, please see Figure 1 for details.
