# Supplementary material for: The clinical characteristics and SARS‐CoV‐2 infection in children of acute hepatitis with unknown aetiology: A meta‐analysis and systematic review
Source: PLoS One. 2024 Dec 5;19(12):e0311772. doi: 10.1371/journal.pone.0311772 (PMC11620374; doi:10.1371/journal.pone.0311772)
Supplement: Attachment — Submitted filename: Response to Reviewers.docx [file pone.0311772.s008.docx]

# Response to Reviewers

1. **Please ensure that your manuscript meets PLOS ONE's style requirements, including those for file naming.**

\#I have changed the manuscript according to PLOS ONE's style.

2. **We note that this manuscript is a systematic review or meta-analysis; our author guidelines therefore require that you use PRISMA guidance to help improve reporting quality of this type of study. Please** **upload copies of the completed PRISMA checklist as Supporting Information with a file name “PRISMA checklist”.**

#I have upload copies of the completed PRISMA checklist as Supporting Information with a file name “PRISMA checklist”.

**3. We noticed you have some minor occurrence of overlapping text with the** following previous publication(s), which needs to be addressed:

https://onlinelibrary.wiley.com/doi/10.1002/jmv.26314
<https://pubmed.ncbi.nlm.nih.gov/35551703/>

#I have cited the above 2 articles, see Ref.26 and Ref.32

**4. We note that your Data Availability Statement is currently as follows: Data availability is not applicable to this article as no new data were created or analyzed in this study.**

#I have upload minimal data set as Supporting Information with a file name “minimal data set”.

5. **Please include** **captions for your Supporting Information files** **at the end of your manuscript, and update any in-text citations to match accordingly.**

#I have upload captions of my Supporting Information at the end of my manuscript.

6. **Please review your** **reference list to ensure that it is complete and correct.**

#Reference list has been checked.
