# Supplementary material for: The clinical characteristics and SARS‐CoV‐2 infection in children of acute hepatitis with unknown aetiology: A meta‐analysis and systematic review
Source: PLoS One. 2024 Dec 5;19(12):e0311772. doi: 10.1371/journal.pone.0311772 (PMC11620374; doi:10.1371/journal.pone.0311772)
Supplement: Attachment — Submitted filename: Response to Reviewers.doc [file pone.0311772.s009.doc]

# Response to Reviewers

1. **Editor's comments:Please add the following sentence and new references to Discussion: "Examining liver enzymes or biomarkers, e.g. ALT, AST, WBC, cytokines and GRP78, may contribute to differential diagnosis, but the specificity issues of these biomarkers should be carefully considered". More references are suggested to cite, with the following ref as examples (citing is optional).**

\#I have added the above sentence and new references to Discussion.see text maked in red.

2. **Reviewer #1: Acute severe hepatitis of unknown aetiology in children presents with a high number of similar cases in a short period of time, and some of them are accompanied by fever, suggesting the possibility of an infectious etiology.**

#I have modified the discussion section based on Review1's suggestion.see text maked in red.
